# A retrospective analysis of submissions, acceptance rate, open peer review operations, and prepublication bias of the multidisciplinary open access journal Head & Face Medicine

**DOI:** 10.1186/1746-160X-3-27

**Published:** 2007-06-11

**Authors:** Thomas Stamm, Ulrich Meyer, Hans-Peter Wiesmann, Johannes Kleinheinz, Murat Cehreli, Zafer C Cehreli

**Affiliations:** 1 Poliklinik für Kieferorthopädie, Universitätsklinikum, Westfälische Wilhelms-Universität, Münster, Germany; 2 Klinik und Poliklinik für Mund-, Kiefer- und Gesichtschirurgie, Heinrich Heine Universität, Düsseldorf, Germany; 3 Klinik und Poliklinik für Mund-, Kiefer- und Gesichtschirurgie, Universitätsklinikum, Westfälische WiIhelms-Universität, Münster, Germany; 4 CosmORAL Oral and Dental Health Polyclinics, Cinnah 7/5 Kavaklýdere, Ankara, Turkey; 5 Department of Pediatric Dentistry, Faculty of Dentistry, Hacettepe University, Ankara, Turkey

## Abstract

**Background:**

*Head & Face Medicine *(HFM) was launched in August 2005 to provide multidisciplinary science in the field of head and face disorders with an open access and open peer review publication platform. The objective of this study is to evaluate the characteristics of submissions, the effectiveness of open peer reviewing, and factors biasing the acceptance or rejection of submitted manuscripts.

**Methods:**

A 1-year period of submissions and all concomitant journal operations were retrospectively analyzed. The analysis included submission rate, reviewer rate, acceptance rate, article type, and differences in duration for peer reviewing, final decision, publishing, and PubMed inclusion. Statistical analysis included Mann-Whitney U test, Chi-square test, regression analysis, and binary logistic regression.

**Results:**

*HFM *received 126 articles (10.5 articles/month) for consideration in the first year. Submissions have been increasing, but not significantly over time. Peer reviewing was completed for 82 articles and resulted in an acceptance rate of 48.8%. In total, 431 peer reviewers were invited (5.3/manuscript), of which 40.4% agreed to review. The mean peer review time was 37.8 days. The mean time between submission and acceptance (including time for revision) was 95.9 days. Accepted papers were published on average 99.3 days after submission. The mean time between manuscript submission and PubMed inclusion was 101.3 days. The main article types submitted to HFM were original research, reviews, and case reports. The article type had no influence on rejection or acceptance. The variable 'number of invited reviewers' was the only significant (p < 0.05) predictor for rejection of manuscripts.

**Conclusion:**

The positive trend in submissions confirms the need for publication platforms for multidisciplinary science. *HFM's *peer review time comes in shorter than the 6-weeks turnaround time the Editors set themselves as the maximum. Rejection of manuscripts was associated with the number of invited reviewers. None of the other parameters tested had any effect on the final decision. Thus, *HFM's *ethical policy, which is based on Open Access, Open Peer, and transparency of journal operations, is free of 'editorial bias' in accepting manuscripts.

**Original data:**

Provided as a downloadable tab-delimited text file (URL and variable code available under section 'additional files').

## Background

Head & Face Medicine (*HFM*) was launched in August 2005 to provide multidisciplinary research with a state-of-the-art publication platform [1-3]. Being clinicians, we realized that the ongoing fragmentation of medical specialties may increase specialist medical knowledge but that any effect of this knowledge on traditional and established therapy strategies is slow. We also realized that, with the increase of new specialties; the borders between the fields had become increasingly blurry. Much important clinical research takes place between different fields, which in turn necessitates a multidisciplinary platform to disseminate the results of research to the relevant audience. However, after a period of one year, no answer could be found to the question as to whether *HFM *will be an ideal platform to disseminate multidisciplinary knowledge in the area of head and face disorders. *HFM *still is developmental in character and the journal's ethical policy based on open access and open peer review results in a commitment to a regular self-analysis of *HFM's *maturation. The aim of the present paper was therefore to evaluate the characteristics of submissions, the effectiveness of the open peer reviewing process, and factors biasing acceptance or rejection of manuscripts. This analysis attempts to generate information to assess the journal's development and was also conducted for the sake of transparency and objectivity in all journal operations of *Head & Face Medicine*.

## Methods

Manuscripts submitted to *Head & Face Medicine *undergo a strictly uniform editorial process. Based on this workflow, the following journal operations were extracted from *HFM*'s Content Management System for submissions between August 2005 and August 2006.

• Number of complete/incomplete submissions

• Date of submission

• Number of reviewers invited

• Number of agreements to review

• Number of reports returned

• Date of report

• Number of acceptances/rejections

• Date of acceptance/rejection

• Date of publishing (provisional)

• Date of PubMed record

The following times were calculated based on the obtained data.

• Peer review time (PRT): The time between date of submission and date when reports are returned to the authors. PRT is at any time greater than the time used for processing the review because of the time differential between invitation to review and agreement of peer reviewers. PRT does not include revision time and re-review time.

• Acceptance/rejection time (A/RT): The time between date of submission and "editorial" acceptance or rejection. A/RT includes revision time and re-review time. Editorial acceptance is different from full acceptance and concerns the content of the paper and positive reports only. Full acceptance is declared when the paper complies with the formatting requirements laid out in the instructions for authors. Full acceptance is, in general, equal to the provisional publication of the article.

• Publishing time (PT): The time between date of submission and date of provisional publication of the paper. With its provisional publication on the HFM website, the paper is immediately accessible via the Internet and searchable by any web browser.

• PubMed availability time (PAT): The time between date of submission and date of inclusion into PubMed of the final title and abstract. The PubMed entry was obtained from the EDAT tag.

Additionally, the following data were evaluated.

• Submission and acceptance rates

• Type of submission

• Editorial workload. Editorial work is difficult to measure. The only quantifiable data are the number of submissions and the number of e-mails generated through communication between authors, reviewers, and editors.

### Statistics

The Mann-Whitney U test was chosen to assess differences in journal operations between accepted and rejected papers. Crosstabs with Chi-square test was used to evaluate differences between various types of articles.

Binary logistic regression analysis was performed to identify variables most responsible for the prediction of acceptance or rejection (editorial bias). For this purpose, the observed event 'editorial decision' was dichotomized to two values, which represent the occurrence (acceptance) or non-occurrence (rejection) of the event.

## Results

### Submission and acceptance rates

**Figure 1:**
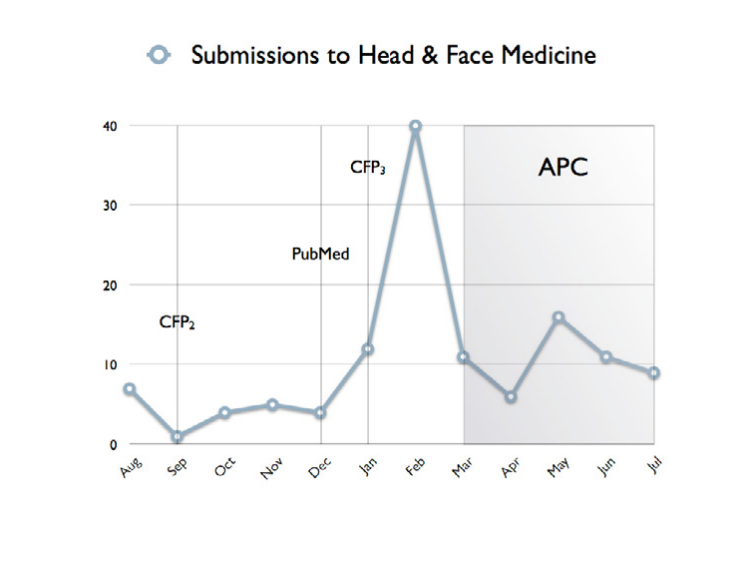
Submissions to *HFM *between August '05 and August '06. The second call for papers to prospective authors was e-mailed in September (CFP_2_) and a further call in January (CFP_3_). PubMed inclusion started on 2^nd ^December. The article processing charge (APC) was introduced in March '06.

In total, 126 manuscripts were submitted between August 2005 and July 2006. An additional number of 40 manuscripts were submitted incomplete and therefore not yet under review. Figure 1 shows a slightly increasing submission rate over the last 12 months with a distinct peak in February, the month before the article processing charge (APC) was introduced. On average, 10.5 articles were submitted per month. Excluding the peak in February (assuming the same number of 12 submissions as in January), the rate would decrease to 8.2 articles per month.

Between August 2005 and August 2006, peer reviewing was completed for 82 articles. Of those, 40 manuscripts were rejected and two were withdrawn, which is equal to an acceptance rate of 48.8%.

### Peer review process

Prospective reviewers for all manuscripts were selected from the Editorial Board and from PubMed only. In total, 431 experts were invited to review 82 manuscripts. 174 peer reviewers agreed to review and 52 of them reviewed more than one paper. 199 invited experts declined to review, while six experts agreed but did not provide any report. The maximum number of invitations sent before two reports were finally received was 18. On average, 5.3 experts were invited per manuscript.

HFM's peer review process is based on a minimum of two reports per manuscript. The peer review time for the first and seond reports were 33.8 and 41.9 days, respectively. In total, the mean PRT was 37.8 days, which comes in shorter than the 6-weeks turnaround time the Editors set themselves as the maximum. The PRT of rejected manuscripts was shorter (35.3 days) when compared to accepted papers (40.3 days), but not to a significant extent (p > 0.05).

The mean acceptance time was 95.9 days. Taking into account the time needed for re-reviews required after authors' revisions, this figure calculates down to approximately 95.9-40.3 = 55.6 days for revision. The mean rejection time was 49 days. The Editors-in-Chief needed approximately 49-35.3 = 13.7 days for the final decision by assessing the reports and manuscripts.

The mean publishing time (PT) was 99.3 days. After this time, the authors' work was first made available to the scientific community because title, abstract, and a provisional PDF of the manuscript were published on the HFM website and thus, became both accessible and retrievable via the Internet. PubMed availability time, the time between submission and inclusion into PubMed of the title and abstract, was on average 101.3 days.

### Type of submission

**Figure 2:**
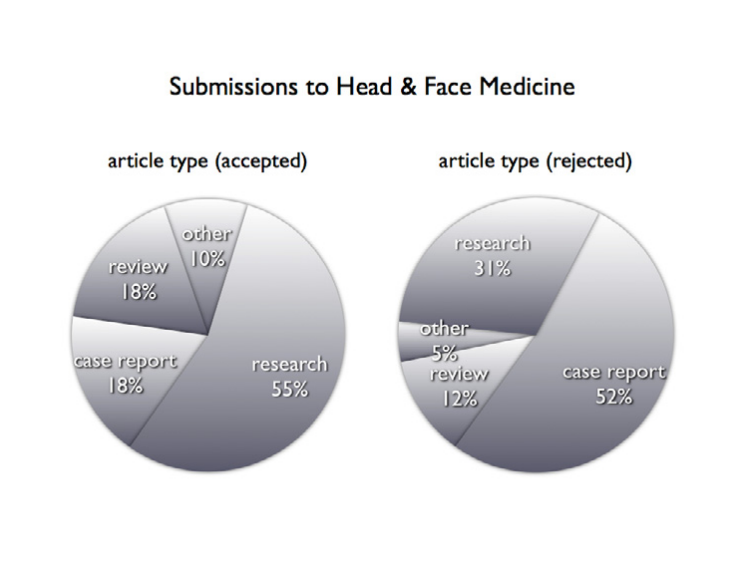
Article types showed significant differences (χ^2^, p < 0.05) when comparing accepted with rejected manuscripts. Case reports were rejected most frequently whereas research papers were accepted more often than other types. In general, 'article type' is not a predictor for rejection or acceptance (p > 0.05).

**Figure 3:**
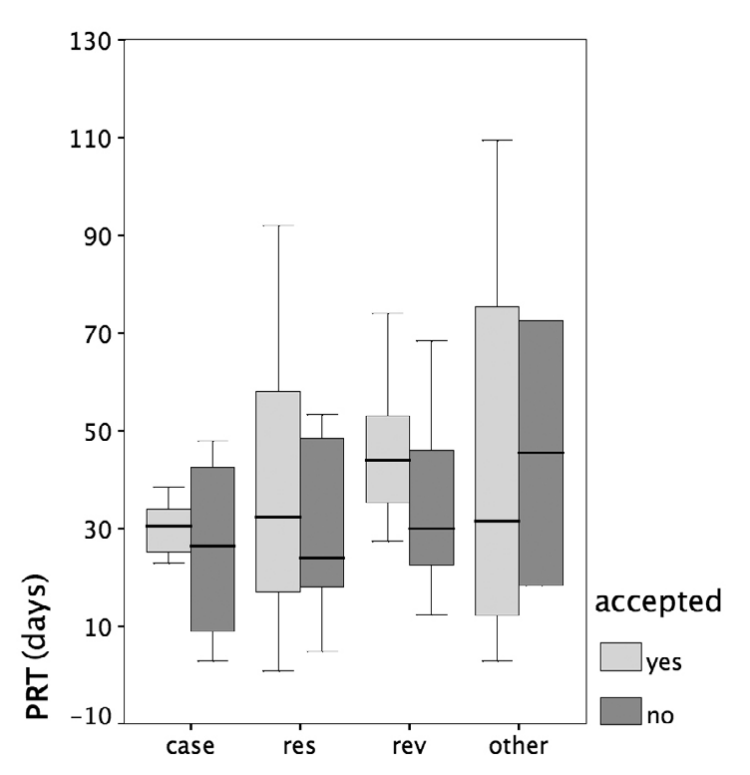
The peer review time (PRT) was not significantly different between article types.

The main article types submitted to *HFM *were original research articles, reviews, and case reports (Figure 2). All other types represented less than 8% of the submitted manuscripts. Research papers were accepted by reviewers most frequently, whereas case reports were rejected more often than other types of articles. Although accepted and rejected papers differed by article type (χ^2^, p < 0.05), there is no increased probability for case reports to be rejected (p > 0.05). In general, the variable 'article type' is not a predictor for rejection or acceptance. There is also no significant relation between article type and the time of peer reviewing (Figure 3). Case reports had a shorter publishing time (p < 0.05) when compared to research papers and reviews.

### Editorial workload

In general, the means of communication used between all parties concerned was e-mail. Only two manuscripts were sent to reviewers by mail or fax. Between August 2005 and August 2006, the *HFM *e-mail account held 2521 e-mails. The correspondence with authors and reviewers comprised 1607 e-mails. 501 of them were exchanged between *BMC *and *HFM *and 413 e-mails were sent to the editorial co-workers. On average, approximately 6.9 e-mails were written per day to ensure the daily editorial workflow. These sums up to 19.7 e-mails per submitted article. Considering the current acceptance rate of 48.8%, an average of 73 e-mails were exchanged for each published article.

**Figure 4:**
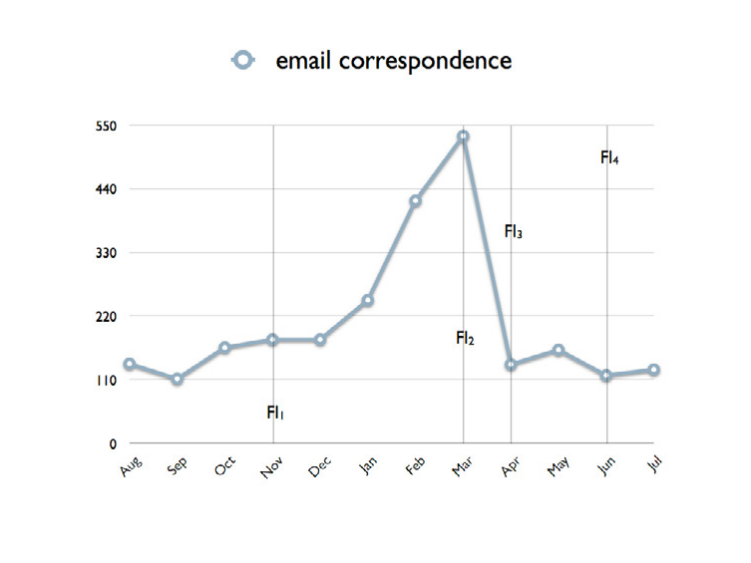
E-mail rate over the last 12 months. Out of 2521 e-mails, 1607 were exchanged between the editors and authors and reviewers. On average, 73 e-mails for each published article were exchanged. Several functionality improvements (FI) facilitated the online peer reviewing process. FI_1 _= e-mail archiving, FI_2 _= elimination of multiple notifications, FI_3 _= accepting articles online, FI_4 _= requesting revisions online.

The e-mail rate in Figure 4 shows a pattern similar to the submission rate (Figure 1). In contrast to the characteristics of the latter one, the correspondence decreased slightly over time. This was due to functionality improvements (FI) of the peer reviewing system. Because there was no handbook at all and the editorial team was unaware of the full functionality of the *BMC *online peer reviewing system, a considerable amount of correspondence was exchanged offline (manually) at the beginning of *HFM*. The first significant improvement (FI_1_) of the online system was the e-mail archiving in November 2005. Any e-mails sent by the editors were then automatically added to a history page, resulting in a chronological overview which facilitates evaluation of the whole peer reviewing process. Because of a further functionality improvement (FI_2_) in March 2006, multiple notifications to authors and reviewers were eliminated. From that point in time, only the following was performed independently from the editorial managemet tools: a) informing authors if revisions were required, b) requesting re-review of a manuscript if required after revisions, and c) accepting a manuscript. This resulted in a significant reduction of e-mails from March to April '06 (Figure 4). Two further improvements were introduced in April and June '06: accepting articles (FI_3_) and requesting revisions online (FI_4_).

### Decision bias

Except for one parameter, none of the obtained variables had an effect on the decision to accept or reject papers. Binary logistic regression revealed a significant relationship (p < 0.05) between rejection of a paper and the number of invited reviewers. The probability of rejection *P*_*R *_could be computed by the logistic equation

PR=1(1+e−z)
 MathType@MTEF@5@5@+=feaafiart1ev1aaatCvAUfKttLearuWrP9MDH5MBPbIqV92AaeXatLxBI9gBaebbnrfifHhDYfgasaacH8akY=wiFfYdH8Gipec8Eeeu0xXdbba9frFj0=OqFfea0dXdd9vqai=hGuQ8kuc9pgc9s8qqaq=dirpe0xb9q8qiLsFr0=vr0=vr0dc8meaabaqaciaacaGaaeqabaqabeGadaaakeaacqWGqbaudaWgaaWcbaGaemOuaifabeaakiabg2da9maalaaabaGaeGymaedabaGaeiikaGIaeGymaeJaey4kaSIaemyzau2aaWbaaSqabeaacqGHsislcqWG6bGEaaGccqGGPaqkaaaaaa@38B6@, where *z *is computed as *z *= *constant *+ *regression coefficient*.

**Figure 5:**
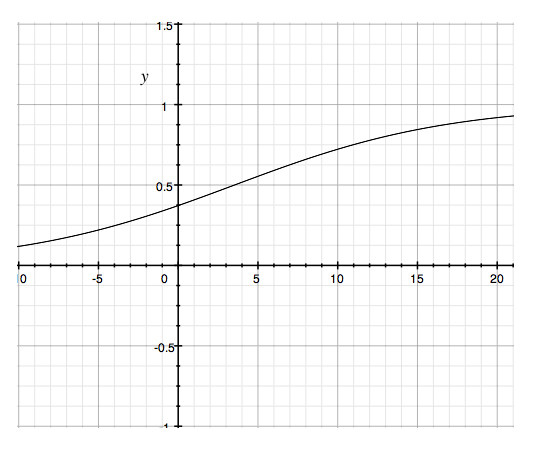
The logistic function produces a sigmoid curve, where y represents the probability of rejection (*P*_*R*_) and x the number of invited reviewers. Inviting a minimum of 2 reviewer corresponds to a probability of rejection *P*_*R *_= 0.44. Inviting 15 reviewers increases *P*_*R *_to 0.85.

Based on our data *z *= -0.519+0.148. The graphical representation is shown in Figure 5. The computed model corresponds to our rejection rate (51.2%). As a mean of 5.3 reviewers were invited, there is a probability of *P*_*R *_= 0.57 that the paper will be rejected.

## Discussion

Medical journals have to assume a high level of ethical responsibility because by disseminating scientific findings they cause far-reaching consequences for patients. Due to the global availability of the Internet, the volume and the speed of dissemination of medical data has grown exponentially. However, such a fortunate consequence for medical science, also puts a strain on control schemes (such as peer reviewing) that are supposed to ensure the quality of the published outcomes.

An important step related to process quality is to reduce pre-publication bias through transparent journal operations. New journals, which cannot rely on a tradition of experience and reputation, therefore have the obligation to demonstrate their process quality and objectivity throughtout the publication process. The obtained data can be useful, furthermore, to assess the profile of other journals. The aim of this paper was therefore to evaluate the characteristics of submissions, the effectiveness of the open-peer reviewing process, and factors biasing acceptance or rejection of manuscripts.

Data on the first-year submission rates to a medical journal are not available. Just as with trans-discipline comparisons, it is uncertain whether this kind of comparison makes sense at all. Despite the difficulties of interpretation, we consider that the slightly increasing submission rate, at a mean 10.5 papers/month in the first year, validates the multidisciplinary approach of HFM. The acceptance rate was established at 48%. The HFM Editorial Team does not consider the number of rejected manuscripts to be a quality criterion for a journal. Thus no comparison was made as to the rejection rates.

The APC introduction in March 2006 seems to have adversely affected the submission rate, which would also explain the high number of submissions in February 2006 (Figure 1). Nevertheless, it is interesting to note, though, that the same trend cannot be observed for the application for waivers. Although the APC can be waived for 35% of all papers (for authors who genuinely have no access to funding) the corresponding application was received in the course of the first year for less than 10% of submitted papers. This fact may be seen as an indicator for lack of information or for a combined effect of funds available and geographic origin of submissions or for the APC's being of secondary importance.

Timely peer reviewing is an exceptionally essential factor for new journals. There seems to be a minimum time when requesting a review from an unpaid, well-renowned reviewer, which it is impossible to shorten any further. Other journals have also recognized that their shortened peer review time could only be achieved at the expense of the destruction of the very process [1]. A mean PRT of 37.8 days could be achieved only by inviting more than two reviewers (5.3 reviewers on average). There was no PRT difference between accepted and rejected papers.

A further important point, besides timely peer reviewing, is fast publication. This time depends on cumulated times of revision, re-reviewing, and the final decision made by the editorial team. The mean acceptance time was 95.9 days, and provisional publication occurred after a mean 99.3 days. This timeframe is the critical item, since it reflects the duration after which the paper becomes retrievable via the Internet for the first time and starts to exist within the scientific community. Another important marker related to this process is the PubMed inclusion. PAT amounted to a mean 101.3 days from submission and depended also on e-publication workflow.

HFM mainly received standard types of manuscripts, such as original research articles, reviews, and case reports. Case studies, database articles, hypotheses, methodology articles, short reports, software articles, and study protocols are underrepresented, indicating an increased need for information to be provided to contributors on the avaliability of publishing different typed of manuscripts in HFM. Although case reports represent the majority of rejected papers (52%), there is no increased probability for this type of article to be rejected according to the logistic regression model. Compared with other article types, the shorter publishing time associated with case reports can be explained by the shorter length of these papers, which also means less revision time. No difference in PRT was found.

The editorial workload is difficult to measure and was presented herein with the e-mail data to describe the amount of editorial time as a basis for comparison. Time is a major factor in the quality of a journal and has to be reasonably supported by staff. Currently, editorial workflow (except peer review) is handled by a core team of 3 editors-in-chief, 1 deputy editor, 1 executive editor, 2 section editors, 2 peer review coordinators, and one statistical advisor.

A hidden decision bias may compromise the objectivity of a journal, and regular analysis is, therefore, required. With the exception of one parameter, none of the recorded journal operations of *HFM *affects significantly the decision as to acceptance or rejection. Only the number of invited peer reviewers was associated with a higher probability of rejection. Inviting a minimum of 2 reviewers corresponds to a probability of rejection *P*_*R *_= 0.44. *HFM*'s reviewer rate of 5.3/manuscript corresponds to a probability of rejection *P*_*R *_= 0.57, which is close to the current rejection rate of 51.2%. Inviting 15 reviewers would increase *P*_*R *_to 0.85. The advantage of keeping the peer review time below 6 weeks is achieved at the expense of inviting more than two reviewers, which in turn increases the probability of rejection.

In our opinion, however, a *P*_*R *_amounting to 0.57 does not indicate a negative effect insofar as one has to take into account that Open Peer generally results in higher acceptance rates [4]. This corresponds to a balance that is indeed confirmed by the lower rejection rate, which amounts to 51.2%.

## Conclusion

The positive trend in submissions to *HFM *confirms the need for publication platforms for multidisciplinary science. *HFM's *peer review time comes in shorter than the 6-weeks turnaround time the Editors set themselves as the maximum. Rejection of manuscripts was associated with the number of invited reviewers but had no negative effect on the overall rejection rate. None of the other parameters had any effect on the final decision. Thus, *HFM's *ethical policy, which is based on Open Access, Open Peer, and transparency of journal operations, was found to be free of 'editorial bias' in accepting manuscripts.

## Competing interests

The authors declare that they have no competing interests other than being Editors-in-Chief of the journal.

## Authors' contributions

TS suggested the original idea for the study, initiated the investigations leading to these results, participated in discussions on the undertaking of the study, did the statistical analysis and interpreted the data, reviewed all iterations of the paper, and wrote the first draft and the final version of the paper. UM interpreted the data, and reviewed and contributed to the writing of all iterations of the paper, including the final version of the manuscript. HPW and JK participated in discussions on the undertaking of the study, interpreted the data, reviewed the paper for content and contributed to the writing of all iterations of the paper, including the final version of the manuscript. MC and ZCC participated in discussions on the undertaking of the study, interpreted the data, reviewed all iterations of the paper and contributed to the writing of the manuscript. MC and ZCC revised the English grammar of the final version of the manuscript. All authors approved the final manuscript.

## Supplementary Material

Additional File 1 Tab-delimited file containing original data. Variables: n = number of article; rev = number of reviewers; peer1 = PRT of reviewer 1; peer2 PRT of reviewer 2; accep = accepted (0 = yes; 1 = no); print = PT; pubmed = PAT; a_p = days between acceptance and print; a_pub = days between acceptance and PubMed inclusion; peer = average peer review time; a_type = article type. Click here for file
